# The Effect of Xanthine Oxidase Inhibitors in the Prevention and Treatment of Stroke: A Systematic Review and Meta-Analysis

**DOI:** 10.3390/jcdd11120409

**Published:** 2024-12-21

**Authors:** Lin Bai, Gerhard Litscher, Xiaoning Li

**Affiliations:** 1 Heilongjiang University of Chinese Medicine, Harbin 150040, China; 2 Swiss University of Traditional Chinese Medicine, SWISS TCM UNI, High-Tech Acupuncture and Digital Chinese Medicine, 5330 Bad Zurzach, Switzerland; 3 President of the International Society for Medical Laser Applications (ISLA Transcontinental), German Vice President of the German-Chinese Research Foundation (DCFG) for TCM, Honorary President of the European Federation of Acupuncture and Moxibustion Societies, Honorary Professor of China Beijing International Acupuncture Training Center, China Academy of Chinese Medical Sciences, Former Head of Two Research Units and the TCM Research Center at the Medical University of Graz, 8053 Graz, Austria; 4 Department of Acupuncture, The Second Affiliated Hospital of Heilongjiang University of Chinese Medicine, Harbin 150006, China

**Keywords:** xanthine oxidase inhibitors, stroke, treatment, prevention, meta-analysis

## Abstract

Background: Xanthine oxidase inhibitors (XOIs) may help prevent stroke. However, heterogeneity can be found in the conclusions of different studies. The relevant evidence was summarized in this systematic review and meta-analysis to further clarify the role of XOIs in the prevention and treatment of stroke, with a focus on evaluating the effects of XOIs in primary and secondary prevention of stroke, acute stroke treatment, and reduction of post-stroke complications. Methods: Randomized controlled trials (RCTs) or cohort studies on the effect of XOIs in the prevention and treatment of stroke were searched in PubMed, EMBASE, and Cochrane Library from inception to 3 March 2024, along with hand searching. The analyses were carried out using Review Manager 5.4. Results: The analysis included 14 studies (115,579 patients). While XOIs did not significantly reduce the risk of stroke (RR: 0.89; 95% CI: 0.59–1.34), they improved post-stroke functional outcomes, with a reduction in the modified Rankin scale scores (mean difference: −0.6; 95% CI: −0.8 to −0.4), decreased intercellular adhesion molecule-1 levels (mean difference: −15.2 ng/mL; 95% CI: −22.3 to −8.1), improved augmentation index (AIx) by 4.2% (95% CI: 2.5–5.9%), reduced central blood pressure (mean reduction: 4.8 mmHg; 95% CI: 2.6–6.9), and delayed carotid intima-media thickness progression (mean difference: −0.05 mm/year; 95% CI: −0.08 to −0.02).

## 1. Introduction

Stroke is the fifth-leading cause of death in the United States, affecting approximately 795,000 people each year, and recurrent stroke accounts for nearly 25% [1]. The mortality of stroke has been further reduced with medical advances, but many patients with stroke are still faced with the risk of severe long-term disability, and more than 50% of patients with stroke require hospitalization for rehabilitation [2]. As a result, an annual direct and indirect medical cost of USD 33 billion is produced by stroke, with the former projected to triple by 2030 [3]. Secondary prevention of stroke involves antihypertensive, lipid-lowering, and hypoglycemic treatments targeting risk factors, along with lifestyle interventions such as smoking cessation, alcohol restriction, appropriate exercise, and healthy diet, thereby reducing the risk of additional ischemic events [4] and improving the post-stroke quality of life of patients. Despite significant progress in the understanding of stroke pathogenesis and drug therapies recommended by guidelines, new cases of stroke are still increasing, both morbidity and mortality of adverse cardiovascular events remain unacceptably high, and there is still a high risk of recurrence, heavy disease burden, and poor prognosis. Therefore, treatment measures require further improvement [5], and other effective methods of primary and secondary prevention of stroke remain to be explored.

Antiplatelet therapy (e.g., aspirin, clopidogrel) and anticoagulant therapy (e.g., warfarin, direct oral anticoagulants) are cornerstone strategies for secondary prevention of stroke. However, additional approaches targeting vascular function and oxidative stress, such as xanthine oxidase inhibitors (XOIs), are being explored to complement these treatments.

The effect of XOIs on cerebrovascular disease has been assessed in many studies [6], and their possible ability to reduce the incidence of stroke and improve prognosis has been found [7], which deserves further study. Ischemia/reperfusion injury following stroke is an inflammatory process characterized by microvascular destruction due to characteristic cell changes. Studies have shown that excessive activation of xanthine oxidase (XO) is the major factor leading to brain dysfunction [8]. Hypoxanthine is produced by the metabolism of purine bases in the human body, which is then catalyzed by XO and converted into uric acid in two steps [8]. During this process, reactive oxygen species (ROS), such as hydrogen peroxide, and O_2-_ are produced, and excess ROS may reduce the production of nitric oxide, leading to endothelial dysfunction (ED) [9]. Characterized by vasoconstriction, platelet activation-induced thrombosis, and smooth muscle proliferation, ED further contributes to atherosclerosis, hypertension, and thrombosis [10]. Moreover, ROS is associated with ischemia/reperfusion injury [11], which may be responsible for the underlying mechanism by which XOIs play a role in reducing the risk of stroke and improving prognoses. Interestingly, uric acid has been explored as a neuroprotective agent in acute ischemic stroke due to its antioxidant properties. This dual role—where uric acid can act as both a pro-inflammatory and an antioxidant molecule—raises questions regarding the use of XOIs, which reduce uric acid levels, in stroke treatment. This paradox warrants further investigation to clarify the net effects of XOIs on stroke outcomes. Despite the potential of XOIs in the treatment of stroke, the existing mechanistic studies are insufficient to assess clinical prognosis, so further studies are required to determine whether XOIs have a protective effect in the prevention and prognosis of stroke. However, relevant clinical epidemiological evidence was previously obtained mainly based on observational studies, and the results were inconsistent and controversial [12]. In recent years, the effect of XOIs has further become a hot spot, with several large randomized controlled trials (RCTs) published. For example, the possible role of XOIs and routine treatment in the prevention and treatment of stroke was compared in the ALL-HEART study [13]. It was found that there was no significant difference in the incidence of stroke events between the XOI group and the routine treatment group (HR = 1.20, 95% CI: 0.89–1.60; *p* = 0.23). In the XILO-FIST study [14], no significant effect of XOIs on white matter hyperintensity (WMH) progression after stroke was observed (95% CI: −0.52–0.17; *p* = 0.33). At present, whether XOIs can reduce the risk of stroke remains unclear, and stroke has not been recognized as an indication for the use of XOIs.

Given the severe impact of stroke on patients’ quality of life and the ongoing burden on the health care system, further research is necessary on drugs able to reduce the incidence of stroke and improve prognoses. XOIs seem to be a therapeutic option. As has been found in a previous systematic review of the effect of XOIs on the prognosis of stroke [15], XOIs seem to yield positive effects on the prognosis of stroke in some ways, such as improving vascular function and reducing disability. However, only the systematic review of secondary prevention of stroke rather than quantitative analyses was carried out, and primary prevention was not discussed. In addition, only five studies were included due to the limited number of original studies previously, and the sample size was small. In this study, therefore, the latest evidence on the effect of XOIs in the prevention and prognosis of stroke was systematically reviewed and quantitatively analyzed through a systematic review and meta-analysis, with more RCTs and cohort studies included than the previous systematic review, and the quality and strength of evidence were assessed to comprehensively investigate the effect of XOIs in primary and secondary prevention of stroke.

## 2. Materials and Methods

### 2.1. Inclusion and Exclusion Criteria

#### 2.1.1. Inclusion Criteria

Inclusion criteria were established based on the PICOS (participants, intervention, comparison, outcome, study design) principles of the Cochrane systematic review. (1) Participants: adults aged above 18 years included for primary prevention, and adults with a history of stroke included for secondary prevention. (2) Intervention: XOIs of any dose. (3) Comparison: placebo or blank control. (4) Outcome: (a) the occurrence of stroke as the endpoint in the studies on the effect of XOIs in prevention; (b) any clinical outcome or improvement in indicators of patients with stroke treated with allopurinol in the studies on the effect of XOIs in treatment. (5) Study design: only RCTs or cohort studies.

#### 2.1.2. Exclusion Criteria

(1) Duplicate publications or studies with similar data; (2) reviews, meta-analyses, systematic reviews, conference reports, case reports, expert experience, and basic experiments; (3) the literature, whose full text was unavailable.

### 2.2. Study Design

This study has been registered with PROSPERO (CRD42023470765). It was conducted using the methodology proposed by the Preferred Reporting Items for Systematic Review and Meta-Analysis Protocols (PRISMA-P) statement [16], and all data used were extracted from individual studies (PRISMA compliance statement: This study was conducted in accordance with the PRISMA guidelines. A PRISMA checklist and flow diagram are provided as Supplementary materials to ensure transparency and rigor in reporting).

### 2.3. Search Strategy

RCTs or cohort studies on the effect of XOIs in the prevention and treatment of stroke were searched in PubMed, EMBASE, and Cochrane Library from inception to 3 March 2024. A second literature search was conducted prior to finalization, with the main search terms of “allopurinol”, “febuxostat”, “xanthine oxidase inhibitor”, “stroke”, “RCT”, and “cohort study”, including their subtitles and synonyms. The search formula is illustrated in the attachment. In addition, the reference lists of identified trials and review articles were carefully checked by a manual search to detect potentially missing eligible studies.

### 2.4. The Literature Screening and Data Extraction

Preliminary screening and a secondary search were carried out by two trained investigators (L.B. and Y.L.) according to the established inclusion and exclusion criteria, so that all published studies were included before finalization. In case of disagreement, a third investigator would participate in the discussion and make a final decision through consultation. Then, the literature retrieved from each database was imported into EndNote X9 to exclude duplicate publications. The literature that did not meet the inclusion criteria was initially excluded by reading the titles and abstracts, and the full text of the remaining literature was read to identify the eligible literature. The information of the eligible literature (basic information such as the first author, year of publication, study site, sample size of the treatment group and control group, gender, age composition, intervention measures, treatment course, outcome indicators, and follow-up time) was extracted using Microsoft Excel. The author would be contacted by email to acquire the missing or unpublished data as far as possible. If data were duplicated across multiple studies, the study with the most comprehensive data was included.

### 2.5. Quality Evaluation

Quality evaluation was conducted by two investigators (L.B. and Y.L.) on the eligible literature by using the revised Cochrane risk of bias tool (RoB2.0) [17]. For the included RCTs, seven items (random sequence generation, allocation concealment, blinding of participants and personnel, blinding of outcome assessment, incomplete outcome data, selective reporting, and other biases) of RoB2.0 were used to categorize the literature as “low risk”, “high risk”, and “unclear risk”. In case of disagreement, a third investigator would participate in the discussion and make a final decision through consultation. Finally, a risk of bias graph was generated.

For the included cohort studies, the Newcastle–Ottawa scale (NOS) [18] was used for quality evaluation. NOS includes eight items in three dimensions (selection of study groups, comparability of groups, and ascertainment of the exposure and outcomes), with a full score of 9 points. NOS ≥ 7 points, =4–6 points, and <4 points were considered high quality, medium quality, and low quality, respectively [19]. Two investigators were responsible for quality evaluation independently. In case of disagreement, they would discuss with a third investigator and make a decision.

### 2.6. Quality of Evidence

The quality of evidence of the included studies was assessed using the GRADE system [20] with five downgrade factors: risk of bias, inconsistency, indirectness, imprecision, and publication bias. Using GRADEpro (McMaster University and Evidence Prime, vers. 2024), the quality of evidence was classified into four grades: high, moderate, low, and very low, indicating the corresponding strength of evidence.

### 2.7. Data Pooling and Analysis

Review Manager 5.4 was used for data analysis. Relative risk (RR) with a corresponding 95% confidence interval (CI) was pooled for binary variables [21]. I2 ≤ 50% suggested no significant heterogeneity among studies, and the data were pooled using the fixed-effects model; I2 > 50% suggested significant heterogeneity among studies, and the random effects model was adopted. The possible sources of heterogeneity were explored by sensitivity analyses using the elimination method. Specifically, each included literature was excluded one by one or a specific group of the literature was excluded, and then the effect size was pooled to check whether the statistical results changed significantly. Funnel plots were created to display the publication bias of outcome indicators involved in ≥10 included studies. Since only five studies were included in the meta-analysis, no publication bias test or funnel plot drawing was carried out.

## 3. Results

### 3.1. Search Results

**Figure 1:**
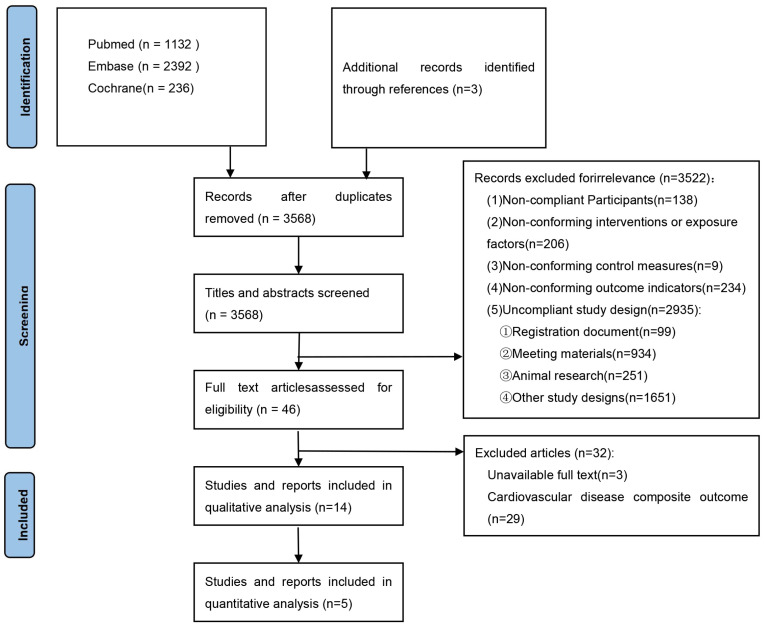
Flow chart of the literature search and inclusion.

Fourteen studies were included in this review [13,14,22,23,24,25,26,27,28,29,30,31,32,33], five of which were included in the meta-analysis [13,23,26,27,28]. The specific procedure of the literature search was displayed in strict accordance with the PRISMA flow chart, as shown in Figure 1.

### 3.2. Characteristics and Quality of Studies

Among the 14 eligible studies included [13,14,22,23,24,25,26,27,28,29,30,31,32,33], there were nine RCTs [34] and five cohort studies [23,24,25,26,27]. The key characteristics and baseline characteristics of patients enrolled in the studies on the effect of XOIs in primary prevention of stroke are shown in Table 1, including two RCTs involving 5834 patients and five retrospective cohort studies involving 109,001 subjects, which compared the effects of allopurinol and blank control/routine treatment on stroke. Furthermore, the key characteristics and baseline characteristics of patients enrolled in the studies on the effect of XOIs in secondary prevention of stroke are shown in Table 2, including seven RCTs involving 744 patients, which compared the effects of allopurinol and blank control/routine treatment on factors related to the prognosis of patients with stroke.

### 3.3. Quality Evaluation of Studies

**Figure 2:**
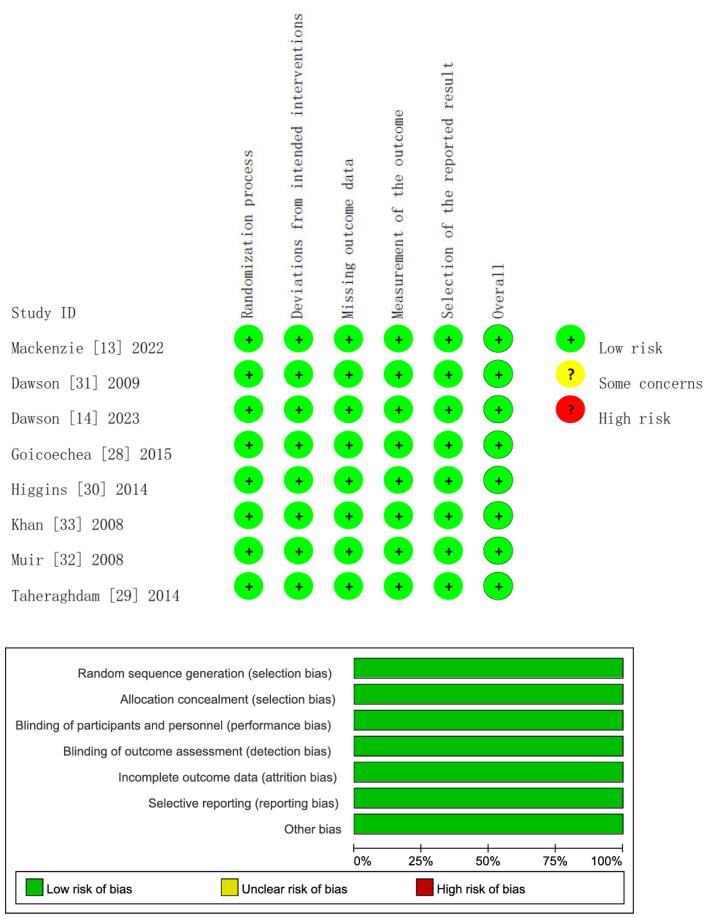
Risk of bias: risk of bias graph and risk of bias summary. All had a low risk of bias (additional explanation: Certain elements categorized as “some concerns” and “unclear risk of bias” (yellow) and “high-risk” (red) are not present in the current context or data set. As a result, they do not apply and cannot be represented in the diagram).

Quality evaluation was carried out on the 14 studies included [13,14,20,21,22,23,24,25,26,27,28,29,30,31] using different tools. To be specific, eight RCTs [13,14,28,29,30,31,32,33] were evaluated for quality using ROB2.0, and it was confirmed that they all had a low risk of bias (Figure 2). The five included cohort studies [23,24,25,26,27] were evaluated for quality using the NOS, and it was found that they all had an NOS score ≥ 7 points, indicating high quality (Table 3).

### 3.4. Results on Primary Prevention

Seven studies [13,23,24,25,26,27,28] were finally included in terms of primary prevention, including two RCTs (low risk) [13,28] and five cohort studies (≥7 points) [23,24,25,26,27], which assessed the incidence of stroke in patients with a history of XOI treatment. A summary of the included studies is provided in Table 4. No significant association between the use of XOIs and decreased incidence of stroke was found in four studies [13,23,27,28]. In RCTs, Mackenzie et al. [13] (low risk), who enrolled elderly people aged 60 years or above, found no significant difference in the incidence of stroke between the allopurinol (600 mg/d) group and the routine treatment group (HR = 1.20, 95% CI: 0.89–1.60; *p* = 0.23); Goicoechea et al. [28] (low risk), who enrolled patients with stable renal function, found no great difference in the incidence of stroke between the allopurinol (100 mg/d) group and the standard treatment group (RR: 0.98; 95% CI: 0.3–3.21). In cohort studies, Ju et al. [23] (NOS: 7 points) included gout patients and found no significant difference in the incidence of stroke events between users (febuxostat 40 mg/80 mg/120 mg, or allopurinol 100 mg/200 mg/300 mg) and non-users of XOIs (HR = 0.821, 95% CI: 0.640–1.054; *p* = 0.121) [21]; Kim et al. [27] (NOS: 8 points) enrolled adults aged 18 years and above and discovered no significant difference in stroke risk between users and non-users of XOIs (HR = 1.01; 95% CI: 0.73–1.41). In another three cohort studies [13,23,27,28], an association between the use of XOIs and a decline in the incidence of stroke was found as follows: Singh et al. [24] (NOS: 8 points), who included patients aged 65 years and above, verified an association between the use of allopurinol and decreased incidence of stroke (HR = 0.91; 95% CI: 0.83–0.99); MacIsaac et al. [25] (NOS: 9 points) enrolled hypertension patients aged 65 years and above and showed that the use of allopurinol was associated with significantly decreased stroke risk (HR = 0.50, 95% CI: 0.32–0.80); Larsen et al. [26] (NOS: 8 points) discovered among patients with hyperuricemia that the use of allopurinol was associated with decreased incidence of stroke (RR: 0.89; 95% CI: 0.81–0.97).

**Figure 3:**
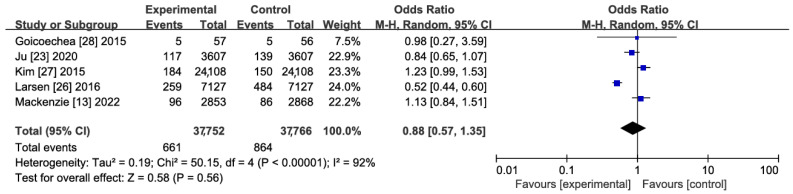
Results regarding primary prevention: occurrence of stroke with pooled analysis of all eligible studies.

**Figure 4:**
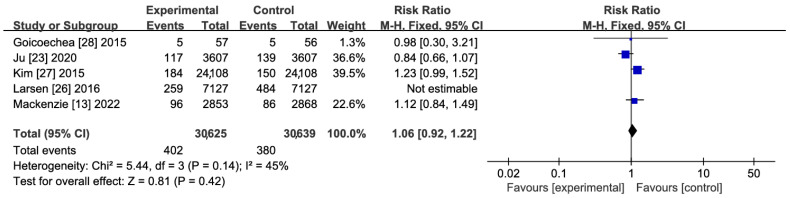
Results regarding primary prevention: occurrence of stroke with forest plot of heterogeneity test results, with Larsen et al.’s study (2016) [26] excluded.

**Figure 5:**
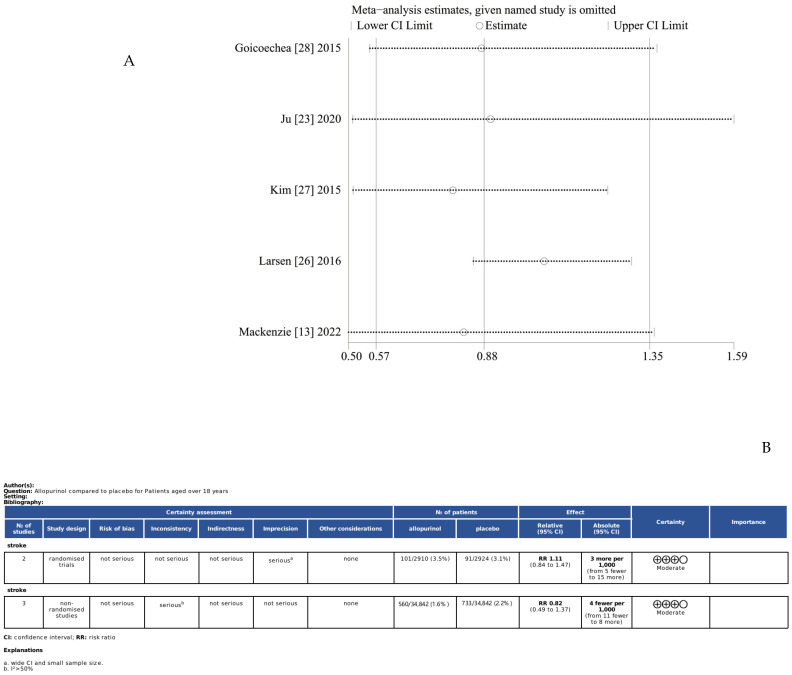
(**A**) Sensitivity analysis of all eligible studies; (**B**) GRADE quality evaluation of all eligible studies.

A total of five studies were included in the quantitative synthesis [13,22,26,27,28], involving 75,518 subjects, including 37,752 in the treatment group and 37,766 in the control group. The results of heterogeneity tests showed *p* = 0.57 and I2 = 92%, suggesting significant heterogeneity across studies, so the random effects model was used for analysis. The effect of XOIs versus blank control/routine treatment on the incidence of stroke was compared using meta-analyses, and no significant association between the use of XOIs and the incidence of stroke was observed (RR: 0.89; 95% CI: 0.59–1.34) (Figure 3). In addition, sensitivity analyses were carried out to explore the possible causes of heterogeneity. Specifically, all included studies were excluded one by one, followed by the pooling of effect sizes. It was found that Larsen et al.’s [26] study resulted in major heterogeneity (*p* = 0.42, I2 = 45%) but it did not affect the robustness of the results, i.e., the incidence of stroke had no statistically significant difference among patients using XOIs compared with the control group (RR: 1.06; 95% CI: 0.92–1.22) (Figure 4 and Figure 5A). It was confirmed by GRADEpro that both RCTs and cohort studies on primary prevention of stroke had moderate quality evidence (Figure 5B).

### 3.5. Results on Secondary Prevention

In the seven RCTs (low risk) [14,22,29,30,31,32,33] included in terms of secondary prevention, the effect of XOIs on clinical endpoints in patients with stroke was not explored, and some protective effects of XOIs in vascular function [33], arteriosclerosis [30], inflammatory response [32], and functional status [29] were found. Higgins et al. [30] (low risk) included patients with recent ischemic stroke or transient ischemic attack (TIA), and found that the patients in the allopurinol (300 mg/d) group had lower central blood pressure (CBP) [−6.6 mmHg (95% CI: −13.0~−0.3), *p* = 0.042] and augmentation index (AIx) [−4.4% (95% CI: −7.9~−1.0), *p* = 0.013], and slower carotid intima-media thickness (CIMT) progression (between-group difference: −0.097 mm, 95% CI: −0.175~−0.019, *p* = 0.015) than in the placebo group. Khan et al. [33] (low risk) found in patients with stroke with hyperuricemia (≥0.38 mmol/L) that the use of allopurinol (300 mg/d) reduced the AIx and improved vascular function compared with the placebo (*p* = 0.031). Muir et al. [32] (low risk) discovered in patients with acute ischemic stroke that the use of allopurinol (300 mg/100 mg) can reduce the increase in intercellular adhesion molecule-1 levels following stroke compared with the placebo (between-group difference: 0.012) [32]. Taheraghdam et al. [29] (low risk) observed among patients with acute ischemic stroke that the use of allopurinol (200 mg/d) was significantly associated with functional status compared with the placebo (OR = 4.646, *p* = 0.014), but it had no significant effects on cerebrovascular reactivity (CVR) [31], cerebral blood pressure variability (BPV) [24], WMH progression, or cognitive improvement [14]. Dawson et al. [14] (low risk) included patients with a history of ischemic stroke or TIA in the last four weeks, and found that compared with the placebo, the use of allopurinol (300 mg) was not significantly associated with WMH progression (between-group difference: −0.17, 95% CI: −0.52–0.17, *p* = 0.33) and cognitive improvement (between-group difference: 0.00, 95% CI: −0.49–0.49, *p* = 0.99). Cognition and WMH progression outcomes were examined with follow-up durations ranging from 12 to 36 months. Due to small sample sizes and limited statistical power, these studies may have been underpowered to detect significant effects. Moreover, on the above basis, MacDonald et al. [22] (low risk) performed a secondary analysis, and found no significant association between the use of allopurinol (300 mg/d) and cerebral BPV (95% CI: 0.31–2.32, *p* = 0.011); Dawson et al. [31] found in patients with a history of subcortical stroke in the last six months that the use of allopurinol (300 mg/d) had no significant association with CVR (95% CI: −13.4–25.5, *p* = 0.64) (Table 5).

## 4. Discussion

A total of 14 studies [13,14,22,23,24,25,26,27,28,29,30,31,32,33] were included in this study, which provided the latest evidence of the effect of XOIs on the incidence and prognosis of stroke. The results revealed that XOIs yielded no significant beneficial effects in the prevention of stroke [13,22,26,27,28]. In terms of secondary prevention, XOIs could ameliorate the prognosis of patients with stroke from vascular function [33], arteriosclerosis [30], inflammatory response [32], and functional status [29], but they had no great impact on CVR [31], BPV [22], WMH progression, or cognitive improvement [14].

The ability of XOIs to reduce oxidative stress, reverse ED, relieve systemic inflammation, and improve arteriosclerosis [34,35,36] has been verified in many previous studies, which may help prevent stroke. This study is the first to summarize whether XOIs can reduce the morbidity of stroke, and it was found that XOIs produced no obvious benefit in preventing stroke. This may be attributed to the dual biological properties of uric acid in cerebrovascular diseases, i.e., uric acid possesses not only pro-inflammatory and pro-atherosclerotic properties, but also strong antioxidant effects [37,38]. XOIs lower uric acid levels and reduce oxidative stress by inhibiting xanthine oxidase, a major source of reactive oxygen species. However, uric acid itself acts as an antioxidant in the brain under certain conditions, which may partially explain the mixed results. This dual role underscores the need to balance oxidative stress reduction with potential neuroprotective effects of uric acid. XOIs act as a mediator by reducing vascular oxidative stress rather than uric acid levels, so the benefit and harm of XOIs for stroke remain inconclusive. Positive results were obtained in three studies [24,25,26], and the possible reason for such heterogeneity is that these three studies included patients older than 65 years old, suggesting that XOIs may benefit some subgroups. However, these three studies were cohort studies with a low evidence grade, so further trials are required in the future.

In terms of secondary prevention, multiple RCTs demonstrated the positive effects of XOIs on vascular function [33], atherosclerosis [30], inflammatory markers, CIMT [32], and functional status [29] in patients with stroke, consistent with the results of previous systematic review [17], and XOIs were also recognized as a promising treatment for improving vascular function, inflammatory markers, and functional status in patients with stroke [17]. In addition, the positive correlations of CIMT, inflammatory factor expression, and degree of arteriosclerosis with increased cerebrovascular mortality and stroke risk have also been verified in previous studies [33,39,40,41,42,43,44]. XOIs can lower the expression of inflammatory adhesion molecules [43], reduce vascular oxidative stress, and ameliorate endothelial function [44], which to some extent provides evidence that allopurinol is beneficial to the prognosis of patients with stroke. People who have experienced ischemic stroke are at risk of vascular event recurrence, progression of cerebrovascular disease, and cognitive decline. However, XOIs have no significant effect on CVR, WMH progression, or cognition of patients with stroke [14,31], possibly because XOIs work mainly by reducing vascular oxidative stress but make less significant improvements in WMH and cognitive decline.

The studies included in this review varied in terms of participants’ age, race, gender composition, baseline comorbidities, and treatment regimens, which could influence the applicability of our results to different populations. Future research should try to eliminate these variations and explore the long-term outcomes of XOIs in specific subpopulations such as the elderly to better understand their potential benefits and risks. Given the potential benefits of XOIs in vascular function and functional recovery, further trials should focus on specific subgroups, such as older adults, patients with hyperuricemia, and those with comorbid cardiovascular disease, to identify those most likely to benefit.

Additionally, exploring the combination of XOIs with other stroke prevention medications, such as antiplatelets or statins, can reveal potential synergistic effects. This area warrants further investigation, as combination therapy may provide a more comprehensive approach to stroke management.

This study also had limitations. The eligible studies included had inconsistencies in the subjects’ demographics and treatment protocols, which could introduce bias. The original studies encompassed both RCTs and cohort studies, which may also contribute to the variability in the results. Subgroup analyses for functional outcomes based on age, comorbidities, and stroke severity were not performed due to limited data. Future studies should consider these factors to better elucidate the effects of XOIs. In addition, dose variability among studies (e.g., 100 mg vs. 300 mg of allopurinol) was not explored. Future meta-analyses and trials should conduct dose–response analyses to determine optimal XOI dosing for vascular and functional outcomes. Future research should strive for more homogeneity in study design and participant characteristics to provide more robust evidence.

## 5. Conclusions

The results of this study, based on high-quality evidence, suggest that XOIs have no significant advantage in reducing the incidence of stroke in primary prevention. However, XOIs are a potentially promising option for ameliorating functional status and vascular function, reducing CBP, and delaying the CIMT progression in patients with stroke. In the future, larger long-term RCTs are needed to further determine the effect of XOIs on patients with stroke. Future research should include the following: (i) longer follow-up periods to assess long-term vascular and cognitive outcomes; (ii) standardized dosing regimens for XOIs to minimize variability; (iii) dose–response analyses to identify optimal therapeutic windows; (iv) combination therapies, such as XOIs with antihypertensive or antiplatelet agents, to explore synergistic effects; (v) stratified analyses based on age, stroke severity, and comorbidities to identify high-risk subgroups.

## Figures and Tables

**Table 1:** Key characteristics of included studies on stroke prevention.

Author	Year	Study Design	Trial Size (Females (%))	Age (Years)	Patients Enrolled	Active Treatment	Comparator	Multi-Center	Follow-Up (Median)	Outcome
Mackenzie [13]	2022	RCT	5721 (24.43)	71.95 (6.75)	Aged 60 years or older, with a history of ischemic heart disease	Allopurinol 600 mg	Usual care	Yes	4.8 years	Stroke
Ju [23]	2020	Retrospective cohort study	13,997 (29.18)	71.79 (14.98)	Patients who had a diagnosis of gout or were prescribed xanthine oxidase inhibitors	Patients who were ever treated by at least one XOI	Patients with no exposure to XOI	Yes	All patients were followed from the index data either on the occurrence of the primary outcome, death, or the last day of search, whichever occurred first.	Stroke
Singh [24]	2016	Retrospective cohort study	28,488 (50.3)	76.5 (7.4)	Patients who were 65 years of age or older and whose new treatment was allopurinol	On allopurino	Not on allopurino	Yes	Ended on the earliest of first date of stroke, losing full Medicare coverage, the date of death, or the end of the study (12/31/12).	Stroke
MacIsaac [25]	2016	Retrospective cohort study	4064 (38.46)	73.09 (5.16)	All patients with hypertension aged ≥65 years	High-dose (≥300 mg daily) or low-dose (<300 mg daily) allopurinol	Not on allopurino	Yes	77.7 months	Stroke
Larsen [26]	2016	Retrospective cohort study	14,254 (27.5)	63.00 (3.07)	Patients with high urate levels (≥6 mg/dL)	on allopurino	Not on allopurino	Yes	5 years	Stroke
Kim [27]	2015	Retrospective cohort study	48,216 (11.95)	51.15 (10.85)	Adult patients aged 18 years or older who had gout.	XOI	Untreated	Yes	1.3 years	Stroke
Goicoechea [28]	2015	RCT	113 (-)	71.75 (8.70)	Patients with eGFRs, 60 mL/min/1.73 m^2^, stable clinical condition (no hospitalizations or cardiovascular events within the 3 months before screening), and stable kidney function	Allopurinol at 100 mg/d	Standard treatment	No	5 years	Stroke

**Table 2:** Key characteristics of included studies on stroke treatment.

Author	Year	Study Design	Trial Size (Females (%))	Age (Years)	Patients Enrolled	Active Treatment	Comparator	Multi-Center	Follow-Up (Median)	Outcome
Dawson [14]	2023	RCT	464 (31.25)	65.7 (8.74)	Patients with a history of ischaemic stroke or transient ischaemic attack (TIA) within the past 4 weeks.	Allopurinol 300 mg twice daily	Placebo	Yes	104 weeks	White matter hyperintensity (WMH) Rotterdam progression score (RPS)
MacDonald [22]	2023	RCT	409 (30.56)	65.90 (8.69)	Patients with a history of ischaemic stroke or transient ischaemic attack (TIA) within the past 4 weeks.	Allopurinol 300 mg twice daily	Placebo	Yes	104 weeks	Blood pressure variability (BPV)
Taheraghdam [29]	2014	RCT	70 (64.29)	68.97 (11.68)	Patients with acute ischemic stroke who had elevated levels of SUA were included.	Allopurinol (200 mg/day)	Placebo	No	3 months	on their functional modified Rankin scale
Higgins [30]	2014	RCT	80 (42.50)	67.9 (9.50)	Patients aged over 18 years with recent ischaemic stroke or TIA.	300 mg allopurinol	Placebo	No	12 months	CBP, arterial stiffness, and CIMT progression
Dawson [31]	2009	RCT	50 (-)	58.3 (10.4)	Individuals with recent (within 6 months) subcortical stroke	300 mg allopurinol	Placebo	No	3 months	Cerebrovascular reactivity (CVR)
Muir [32]	2008	RCT	50 (64.00)	70 (13)	Patients with acute ischemic stroke	Allopurinol (high-(300 mg once a day) or low-(100 mg once a day))	Placebo	No	6 weeks	Circulating inflammatory markers
Khan [33]	2008	RCT	30 (21)	68.45 (8.07)	Thirty survivors of stroke with high serum urate (≥0.38 mmol/L—the local upper limit of normal)	300 mg allopurinol	Placebo	No	8 weeks	Arterial wave reflection, determined from the augmentation index (AIx).

**Table 3:** Evaluation results by NOS.

Study	Selection				Comparability	Outcome	Follow-Up		Total Score
	Representative of the Exposed Cohort	Selection of the Nonexposed Cohort	Ascertainment of Exposure	Demonstration That Outcome of Interest Was Not Present at the Start of the Study	Comparability of Cohorts on the Basis of the Design or Analysis	Assessment of Outcome	Was Follow-Up Long Enough for Outcomes to Occur?	Adequacy of Follow-Up of Cohorts	
Ju [23] 2020	★	★	★	★	★☆	★	☆	★	7
Singh [24] 2016	★	★	★	★	★★	★	★	★	9
MacIsaac [25] 2016	★	★	★	★	★☆	★	★	★	8
Larsen [26] 2016	★	★	★	★	★☆	★	★	★	8
Kim [27] 2015	★	★	★	★	★☆	★	★	★	8

★: one point is added, ☆: the point is not awarded (★: 1; ☆: 0).

**Table 4:** Summary of systematic review results regarding primary prevention.

Study	Region	Year	Study Design	Trial Size	Follow-Up (Median)	Patients Enrolled	Intervention	Comparison	Outcome	Main Conclusions	Risk-of-Bias Judgment/Quality Score
Mackenzie [13] 2022	England	2022	RCT	5721	4.8 years	Aged 60 years or older, with a history of ischemic heart disease	Allopurinolto (at 600 mg/d or 300 mg/d)	Usual care	Stroke	No significant difference was observed between XOI users and XOI non-users for stroke events (HR = 1.20, 95%CI, 0.89–1.60; *p* = 0.23)	Low
Ju [23] 2020	Hong Kong	2020	Retrospective cohort study	13,997	All patients were followed from the index data either on the occurrence of the primary outcome, death, or the last day of the search, whichever occurred first	Patients who had a diagnosis of gout or were prescribed xanthine oxidase inhibitors	XOI Among the XOI user cohort, febuxostat users (febuxost at 40 mg/80 mg/12 mg daily) and allopurinol users (allopurinol 100 mg/200 mg/300 mg daily)	No XOI	Stroke	No significant difference was observed between XOI users and XOI non-users for stroke events (HR = 0.821, 95% CI, 0.640–1.054; *p* = 0.121)	7
Singh [24] 2016	USA	2016	Retrospective cohort study	28,488	Ended on the earliest of first date of stroke, losing full Medicare coverage, the date of death, or the end of the study (12/31/12)	Patients who were 65 years of age or older and whose new treatment was allopurinol	Allopurino	Not on allopurino	Stroke	Allopurinol use was associated with 9% lower hazard ratio for stoke, 0.91 (95% CI, 0.83 to 0.99). Compared with no allopurinol use, allopurinol use durations of 181 days to 2 years, 0.88 (95% CI, 0.78 to 0.99) and >2 years, 0.79 (95% CI, 0.65 to 0.96) were significantly associated with lower multivariable-adjusted hazard of stroke. In subgroup analyses, significant associations were noted between allopurinol use and the risk of ischemic stroke, 0.89 (95% CI, 0.81 to 0.98); associations were not significant for hemorrhagic stroke, 1.01 (95% CI, 0.79 to 1.29). Age 75–85 and ≥85, black race, higher Charlson–Romano index score and the use of beta-blockers were associated with higher hazards of incident stroke.	9
MacIsaac [25] 2016	United Kingdom	2016	Retrospective cohort study	4064	77.7 months	All patients with hypertension aged ≥65 years	High-dose (≥300 mg daily) or low-dose (<300 mg daily) allopurinol	Not on allopurino	Stroke	A total of 2032 patients exposed to allopurinol- and 2032 matched patients who were nonexposed were studied. Allopurinol use was associated with a significantly lower risk of both stroke (hazard ratio, 0.50; 95% confidence interval, 0.32–0.80) than patients who were nonexposed in the control. In patients who were exposed, high-dose treatment with allopurinol (n = 1052) was associated with a significantly lower risk of stroke (hazard ratio, 0.58; 95% confidence interval, 0.36–0.94) than low-dose treatment (n = 980).	8
Larsen [26] 2016	Denmark	2016	Retrospective cohort study	14,254	5 years	Patients with high urate levels (≥6 mg/dL)	On allopurino	Not on allopurino	stroke	There were 14,254 participants (7127 in the allopurinol group and 7127 in the control group) and 259 patients treated with allopurinol experienced stroke events compared with 484 in the control group (RR, 0.54; 95% CI, 0.46–0.62).	8
Kim [27] 2015	USA	2015	Retrospective cohort study	48,216	1.3 years	Adult patients aged 18 years or older who had gout	XOI	Untreated	Stroke	In the primary as-treated analysis, XOI initiation was not associated with the risk of stroke(HR, 1.01; 95%CI, 0.73–1.41). No significant difference was observed between XOI users and XOI non-users for stroke events.	8
Goicoechea [28] 2015	Spain	2015	RCT	113	82.45 months	Ppatients with eGFRs, 60 mL/min/1.73 m^2^, stable clinical condition (no hospitalizations or cardiovascular events within the 3 months before screening), and stable kidney function	Allopurinol at 100 mg/d	Standard treatment	Stroke	There were 113 participants (57 in the allopurinol group and 56 in the control group) and 5 patients treated with allopurinol experienced stroke events compared with 5 in the control group (RR, 0.98; 95% CI, 0.30–3.21). No significant difference was observed between XOI users and XOI non-users for stroke events.	Low

**Table 5:** Summary of systematic review results regarding secondary prevention.

Study	Region	Year	Study Design	Trial Size	Follow-Up (Median)	Patients Enrolled	Intervention	Comparison	Outcome	Main Conclusions	Risk-of-Bias Judgment
Dawson [14] 2023	United Kingdom	2023	RCT	464	104 weeks	Patients with a history of ischaemic stroke or transient ischaemic attack (TIA) within the past 4 weeks.	Allopurinol 300 mg twice daily	Placebo	White matter hyperintensity (WMH) Rotterdam progression score (RPS)	A total of 372 participants (189 with placebo and 183 with allopurinol) attended for week 104 MRI and were included in the analysis of the primary outcome. The RPS at week 104 was 1.3 (SD 1.8) with allopurinol and 1.5 (SD 1.9) with placebo (between group difference −0.17, 95% CI −0.52 to 0.17, *p* = 0.33). The change in the MOCA score at week 104 was between-group difference 0.00, 95% CI −0.49 to 0.49, *p* = 0.99.	Low
MacDonald [22] 2023	United Kingdom	2023	RCT	409	104 weeks	Patients with a history of ischaemic stroke or transient ischaemic attack (TIA) within the past 4 weeks.	Allopurinol 300 mg twice daily	Placebo	Blood pressure variability (BPV)	196 participants were included in analyses of short-term BPV at week 4. Two measures were reduced by allopurinol: the standard deviation (SD) of systolic BP (by 1.30 mmHg (95% confidence interval (CI) 0.18–2.42, *p* = 0.023)) and the average real variability (ARV) of systolic BP (by 1.31 mmHg (95% CI 0.31–2.32, *p* = 0.011)). There were no differences in other measures at week 4 or in any measure at 2 years. Allopurinol is unlikely to lead to an important reduction in BPV in people with ischemic stroke or TIA.	Low
Taheraghdam [29] 2014	Iran	2014	RCT	70	3 months	Patients with acute ischemic stroke who had elevated levels of SUA were included.	Allopurinol (200 mg/day)	Placebo	On their functional modified Rankin scale	The final favorable functional status (mRS = 0–2) was 23 (65.7%) and 14 (40.0%) in the treated and placebo groups, respectively, which was strongly associated with allopurinol consumption (OR = 4.646, *p* = 0.014) and age ≤70 years (OR = 0.139, *p* = 0.005) in patients with ischemic stroke after adjusting for confounders. There was no significant difference in death between allopurinol-treated cases (3; 8.6%) and placebo-treated ones (6; 17.2%; *p* = 0.278). Allopurinol treatment was well tolerated and improved the 3-month functional status of patients with acute ischemic stroke who had high levels of SUA.	Low
Higgins [30] 2014	Glasgow	2014	RCT	50	12 months	Patients aged over 18 years with recent ischaemic stroke or TIA	Allopurinol 300 mg	Placebo	CBP, arterial stiffness, and CIMT progression	Systolic CBP [−6.6 mm Hg (95% CI−13.0 to−0.3), *p* = 0.042] and augmentation index [−4.4% (95%CI −7.9 to−1.0), *p* = 0.013] were each lower following allopurinol treatment compared with placebo at 12 months. Progression in mean common CIMT at 1 year was less in allopurinol-treated patients compared with placebo [between-group difference [−0.097 mm (95%CI −0.175 to −0.019), *p* = 0.015]. No difference was observed for measures of endothelial function. Allopurinol lowered CBP and reduced CIMT progression at 1 year compared with placebo in patients with recent ischaemic stroke and TIA.	Low
Dawson [31] 2009	Glasgow	2009	RCT	80	3 months	Individuals with recent (within 6 months) subcortical stroke	300 mg allopurinol	Placebo	Cerebrovascular reactivity (CVR)	CVR did not change following treatment with allopurinol [median CVR change 0.89% after allopurinol (n = 20) and −0.68% after placebo (n = 25); 95% confidence interval for estimated difference in medians −13.4, 25.5, *p* = 0.64].	Low
Muir [32] 2008	Glasgow	2008	RCT	50	6 weeks	Patients with acute ischemic stroke	Allopurinol (high-(300 mg once a day) or low-(100 mg once a day))	Placebo	Circulating inflammatory markers	Intercellular adhesion molecule-1 concentration (ng/mL) rose by 51.2 in the placebo group, rose slightly (by 10.6) in the low-dose allopurinol group, but fell in the high-dose group (by 2.6; difference between groups *p* = 0.012, Kruskal–Wallis test). Allopurinol treatment attenuated the rise in intercellular adhesion molecule-1 levels seen after stroke.	Low
Khan [33] 2008	Scotland	2008	RCT	30	8 weeks	Survivors of stroke with high serum urate (≥0.38 mmol/L—the local upper limit of normal)	300 mg allopurinol	Placebo	Arterial wave reflection, determined from the augmentation index (AIx).	For patients treated with allopurinol, there was a reduction in AIx from 26.08 ± 3.31% to 20.15 ± 2.23% compared with an increase in the placebo group from 23.57 ± 3.13% to 27.64 ± 3.44% (*p* = 0.031, ANOVA).	Low

## Data Availability

The data presented in this study are available on request from the first author.

## Supplementary Materials

The following supporting information can be downloaded at: https://www.mdpi.com/article/10.3390/jcdd11120409/s1.

## Abbreviations

XOIs—xanthine oxidase inhibitors; RCTs—randomized controlled trials; CBP—central blood pressure; CIMT—carotid intima-media thickness; ROS—reactive oxygen species; ED—endothelial dysfunction; WMH—white matter hyperintensity; CVR—cerebrovascular reactivity; BPV—blood pressure variability.

## References

[1] Xia Y., Liu H., Zhu R. (2023). Risk factors for stroke recurrence in young patients with first-ever ischemic stroke: A meta-analysis. World J. Clin. Cases.

[2] Chan B.P.L., Wong L.Y.H., Tan B.Y.Q., Yeo L.L.L., Venketasubramanian N. (2024). Dual antiplatelet therapy for the acute management and long-term secondary prevention of ischemic stroke and transient ischemic attack, an updated review. J. Cardiovasc. Dev. Dis..

[3] Kleindorfer D.O., Towfighi A., Chaturvedi S., Cockroft K.M., Gutierrez J., Lombardi-Hill D., Kamel H., Kernan W.N., Kittner S.J., Leira E.C. (2021). Guideline for the Prevention of Stroke in Patients with Stroke and Transient Ischemic Attack: A Guideline from the American Heart Association/American Stroke Association. Stroke.

[4] Li L., Scott C.A., Rothwell P.M. (2022). Association of younger vs. older ages with changes in incidence of stroke and other vascular events, 2002–2018. JAMA.

[5] Bhatia K., Ladd L.M., Carr K.H., Di Napoli M., Saver J.L., McCullough L.D., Farahabadi M.H., Alsbrook D.L., Hinduja A., Garcia J.G.O. (2023). Contemporary antiplatelet and anticoagulant therapies for secondary stroke prevention: A narrative review of current literature and guidelines. Curr. Neurol. Neurosci. Rep..

[6] Maruhashi T., Higashi Y., Yoshida H., Tanaka A., Eguchi K., Tomiyama H., Kario K., Kato T., Oda N., Tahara N. (2022). Long-term effect of febuxostat on endothelial function in patients with asymptomatic hyperuricemia: A sub-snalysis of the PRIZE study. Front. Cardiovasc. Med..

[7] Saito Y., Tanaka A., Koide Y., Yoshida H., Uchida D., Matsunaga K., Yokota N., Ueyama C., Kobayashi Y., Node K. (2022). Impact of febuxostat on visit-to-visit blood pressure variability: Insights from the randomised PRIZE Study. RMD Open.

[8] Maciejczyk M., Nesterowicz M., Zalewska A., Biedrzycki G., Gerreth P., Hojan K., Gerreth K. (2022). Salivary Xanthine Oxidase as a potential biomarker in stroke diagnostics. Front. Immunol..

[9] Heinig M., Johnson R.J. (2006). Role of uric acid in hypertension, renal disease, and metabolic syndrome. Clevel. Clin. J. Med..

[10] Zhang J., Dierckx R., Mohee K., Clark A.L., Cleland J.G. (2017). Xanthine oxidase inhibition for the treatment of cardiovascular disease: An updated systematic review and meta-analysis. ESC Heart Fail..

[11] Bredemeier M., Lopes L.M., Eisenreich M.A., Hickmann S., Bongiorno G.K., d’Avila R., Morsch A.L.B., Stein F.D.S., Campos G.G.D. (2018). Xanthine oxidase inhibitors for prevention of cardiovascular events: A systematic review and meta-analysis of randomized controlled trials. BMC Cardiovasc. Disord..

[12] Liuzzo G., Patrono C. (2023). Allopurinol does not improve cardiovascular outcomes in ischaemic heart disease. Eur. Heart J..

[13] Mackenzie I.S., Hawkey C.J., Ford I., Greenlaw N., Pigazzani F., Rogers A., Struthers A.D., Begg A.G., Wei L., Avery A.J. (2022). Allopurinol versus usual care in UK patients with ischaemic heart disease (ALL-HEART): A multicentre, prospective, randomised, open-label, blinded-endpoint trial. Lancet.

[14] Dawson J., Robertson M., Dickie D.A., Bath P., Forbes K., Quinn T., Broomfield N.M., Dani K., Doney A., Houston G. (2023). Xanthine oxidase inhibition and white matter hyperintensity progression following ischaemic stroke and transient ischaemic attack (XILO-FIST): A multicentre, double-blinded, randomised, placebo-controlled trial. eClinicalMedicine.

[15] Britnell S.R., Chillari K.A., Brown J.N. (2018). The role of xanthine oxidase inhibitors in patients with history of stroke: A systematic review. Curr. Vasc. Pharmacol..

[16] Moher D., Shamseer L., Clarke M., Ghersi D., Liberati A., Petticrew M., Shekelle P., Stewart L.A. (2015). Preferred reporting items for systematic review and meta-analysis protocols (PRISMA-P) 2015 statement. Syst. Rev..

[17] Sterne J.A.C., Savović J., Page M.J., Elbers R.G., Blencowe N.S., Boutron I., Cates C.J., Cheng H.Y., Corbett M.S., Eldridge S.M. (2019). RoB 2: A revised tool for assessing risk of bias in randomised trials. BMJ.

[18] Stang A. (2010). Critical evaluation of the Newcastle-Ottawa scale for the assessment of the quality of nonrandomized studies in meta-analyses. Eur. J. Epidemiol..

[19] Hartling L., Milne A., Hamm M.P., Vandermeer B., Ansari M., Tsertsvadze A., Dryden D.M. (2013). Testing the Newcastle Ottawa Scale showed low reliability between individual reviewers. J. Clin. Epidemiol..

[20] Schünemann H.B.J., Guyatt G., Oxman A. (2013). GRADE Handbook for Grading Quality of Evidence and Strength of Recommendations. The GRADE Working Group. https://www.teachepi.org/wp-content/uploads/OldTE/documents/courses/GRADE_Workshop_Flyer_Final.pdf.

[21] Higgins J.P., Thompson S.G. (2002). Quantifying heterogeneity in a meta-analysis. Stat. Med..

[22] MacDonald A.S., McConnachie A., Dickie D.A., Bath P.M., Forbes K., Quinn T., Broomfield N.M., Dani K., Doney A., Muir K.W. (2024). Allopurinol and blood pressure variability following ischemic stroke and transient ischemic attack: A secondary analysis of XILO-FIST. J. Hum. Hypertens..

[23] Ju C., Lai R.W.C., Li K.H.C., Hung J.K.F., Lai J.C.L., Ho J., Liu Y., Tsoi M.F., Liu T., Cheung B.M.Y. (2020). Comparative cardiovascular risk in users versus non-users of xanthine oxidase inhibitors and febuxostat versus allopurinol users. Rheumatology.

[24] Singh J.A., Yu S. (2016). Allopurinol and the risk of stroke in older adults receiving medicare. BMC Neurol..

[25] MacIsaac R.L., Salatzki J., Higgins P., Walters M.R., Padmanabhan S., Dominiczak A.F., Touyz R.M., Dawson J. (2016). Allopurinol and cardiovascular outcomes in adults with hypertension. Hypertension.

[26] Larsen K.S., Pottegård A., Lindegaard H.M., Hallas J. (2016). Effect of allopurinol on cardiovascular outcomes in hyperuricemic patients: A cohort study. Am. J. Med..

[27] Kim S.C., Schneeweiss S., Choudhry N., Liu J., Glynn R.J., Solomon D.H. (2015). Effects of xanthine oxidase inhibitors on cardiovascular disease in patients with gout: A cohort study. Am. J. Med..

[28] Goicoechea M., Garcia de Vinuesa S., Verdalles U., Verde E., Macias N., Santos A., de Jose A.P., Cedeño S., Linares T., Luño J. (2015). Allopurinol and progression of CKD and cardiovascular events: Long-term follow-up of a randomized clinical trial. Am. J. Kidney Dis..

[29] Taheraghdam A.A., Sharifipour E., Pashapour A., Namdar S., Hatami A., Houshmandzad S., Sadeghihokmabadi E., Tazik M., Rikhtegar R., Mahmoodpoor A. (2014). Allopurinol as a preventive contrivance after acute ischemic stroke in patients with a high level of serum uric acid: A randomized, controlled trial. Med. Princ. Pract. Int. J. Kuwait Univ. Health Sci. Cent..

[30] Higgins P., Walters M.R., Murray H.M., McArthur K., McConnachie A., Lees K.R., Dawson J. (2014). Allopurinol reduces brachial and central blood pressure, and carotid intima-media thickness progression after ischaemic stroke and transient ischaemic attack: A randomised controlled trial. Heart.

[31] Dawson J., Quinn T.J., Harrow C., Lees K.R., Walters M.R. (2009). The effect of allopurinol on the cerebral vasculature of patients with subcortical stroke; A randomized trial. Br. J. Clin. Pharmacol..

[32] Muir S.W., Harrow C., Dawson J., Lees K.R., Weir C.J., Sattar N., Walters M.R. (2008). Allopurinol use yields potentially beneficial effects on inflammatory indices in those with recent ischemic stroke: A randomized, double-blind, placebo-controlled trial. Stroke.

[33] Khan F., George J., Wong K., McSwiggan S., Struthers A.D., Belch J.J.F. (2008). Allopurinol treatment reduces arterial wave reflection in stroke survivors. Cardiovasc. Ther..

[34] Farquharson C.A., Butler R., Hill A., Belch J.J., Struthers A.D. (2002). Allopurinol improves endothelial dysfunction in chronic heart failure. Circulation.

[35] Qazi S.U., Qamar U., Maqsood M.T., Gul R., Ansari S.A., Imtiaz Z., Noor A., Suheb M.Z.K., Zaheer Z., Andleeb A. (2023). Efficacy of allopurinol in improving endothelial dysfunction: A systematic review and meta-analysis. High. Blood Press. Cardiovasc. Prev..

[36] Aziz N., Jamil R.T. (2024). Biochemistry, Xanthine Oxidase. StatPearls.

[37] Qin S., Xiang M., Gao L., Cheng X., Zhang D. (2024). Uric acid is a biomarker for heart failure, but not therapeutic target: Result from a comprehensive meta-analysis. ESC Heart Fail..

[38] Gupta M.K., Singh J.A. (2019). Cardiovascular disease in gout and the protective effect of treatments including urate-lowering therapy. Drugs.

[39] Flach C., Muruet W., Wolfe C.D.A., Bhalla A., Douiri A. (2020). Risk and secondary prevention of stroke recurrence: A population-base cohort study. Stroke.

[40] Hillen T., Coshall C., Tilling K., Rudd A.G., McGovern R., Wolfe C.D. (2003). Cause of stroke recurrence is multifactorial: Patterns, risk factors, and outcomes of stroke recurrence in the South London Stroke Register. Stroke.

[41] Yen F.S., Hsu C.C., Li H.L., Wie J.C., Hwu C.M. (2020). Urate-lowering therapy may mitigate the risks of hospitalized stroke and mortality in patients with gout. PLoS ONE.

[42] Miah R., Fariha K.A., Sony S.A., Ahmed S., Hasan M., Mou A.D., Barman Z., Hasan A., Mohanto N.C., Ali N. (2022). Association of serum xanthine oxidase levels with hypertension: A study on Bangladeshi adults. Sci. Rep..

[43] Kinugasa Y., Ogino K., Furuse Y., Shiomi T., Tsutsui H., Yamamoto T., Igawa O., Hisatome I., Shigemasa C. (2003). Allopurinol improves cardiac dysfunction after ischemia-reperfusion via reduction of oxidative stress in isolated perfused rat hearts. Circ. J..

[44] Dawson J., Quinn T., Walters M. (2007). Uric acid reduction: A new paradigm in the management of cardiovascular risk?. Curr. Med. Chem..

